# Interplay of Biochemical, Genetic, and Immunohistochemical Factors in the Etio-Pathogenesis of Gastric Ulcer in Rats: A Comparative Study of the Effect of Pomegranate Loaded Nanoparticles Versus Pomegranate Peel Extract

**DOI:** 10.3389/fphys.2021.649462

**Published:** 2021-03-23

**Authors:** Nessren M. Abd el-Rady, Marwa A. Dahpy, Amel Ahmed, Dalia A. Elgamal, Safy Hadiya, Mai A. M. Ahmed, Zain El-Abdeen Ahmed Sayed, Doaa Abdeltawab, Ahmed Shawkat Abdelmohsen, Alshaimaa Abdelkhaliq Mohammad Farrag, Ahmed M. Ashmawy, Marwa K. Khairallah, Heba M. Galal

**Affiliations:** ^1^Medical Physiology Department, Faculty of Medicine, Assiut University, Assiut, Egypt; ^2^Medical Physiology Department, Sphinx University, New Assiut, Egypt; ^3^Medical Biochemistry and Molecular Biology Department, Faculty of Medicine, Assiut University, Assiut, Egypt; ^4^Department of Histology and Cell Biology, Faculty of Medicine, Assiut University, Assiut, Egypt; ^5^Assiut International Center of Nanomedicine, Al-Rajhy Liver Hospital, Assiut University, Assiut, Egypt; ^6^Department of Pharmcognosy and Natural Products Chemistry, Faculty of Pharmacy, Assiut University, Assiut, Egypt; ^7^Department of Internal Medicine and Gastroenterology, Gastroenterology and Hepatology Unit, Assiut University Hospital, Assiut, Egypt; ^8^Tropical Medicine and Gastroenterology Department, Alrajhi Hospital, Assiut University, Assiut, Egypt; ^9^Department of Anatomy, College of Medicine, University of Bisha, Bisha, Saudi Arabia; ^10^Nephrology and Internal Medicine, Assiut University, Faculty of Medicine, Assiut University, Assiut, Egypt; ^11^Department of Medical Physiology, College of Medicine, Jouf University, Sakaka, Saudi Arabia

**Keywords:** IND-induced gastric ulcer, pomegranate loaded nanoparticles, nitric oxide, TAC, TNF-α, PGE_2_, IL

## Abstract

**Background:**

Few data are available about the role of herbal extract loaded nanoparticles as an alternative safe medicine for the management of a gastric ulcer.

**Aim:**

This work is targeted at exploring the physiological effects of pomegranate loaded nanoparticles (PLN) against an indomethacin IND-induced gastric ulcer and comparing the results with traditional pomegranate peel extract (PPE).

**Methods:**

Twenty-four rats were equally distributed into four groups: control, IND-treated, PLN-treated, and PPE-treated groups. Gross examination of gastric mucosa, and the calculation of ulcer and inhibition indices were done. Serum malondialdehyde (MDA), total antioxidant capacity (TAC), interleukin 2 (IL-2), IL-6, IL-10, gastric homogenate prostaglandin E_2_ (PGE_2_), and nitric oxide (NO) were estimated. Mucosal endothelial nitric oxide synthase (eNOS mRNA) expression was identified by qPCR. Histological and immuno-histochemical staining of Tumor necrosis factor-α (TNF-α) and eNOS of stomach mucosa were performed.

**Results:**

In comparison with the control group, IND-treated rats showed visible multiple ulcers with ulcer index, serum MDA, IL-2 and IL-6 were elevated while IL-10, PGE_2_, NO, and eNOS mRNA expression were significantly reduced. Damaged surface epithelium with disrupted glandular architecture and heavy leucocyte infiltration of lamina propria was noticed. Immunohistochemical staining of stomach mucosa revealed marked increased TNF-α and reduced eNOS. Oral administration of PLN and PPE succeeded in improving the gross mucosal picture, and all biochemical, histological, and immunohistochemical alterations.

**Conclusion:**

Both PLN and PPE potently alleviated IND-induced gastric ulceration *via* increasing TAC, PGE_2_, NO, eNOS mRNA, and protein expression. However, the healing effect of PLN was obviously greater than PPE-treated rats.

## Introduction

A peptic ulcer is an inflammatory discontinuity in the lining of the gastrointestinal tract, usually in the stomach and duodenum. It is the most common form of gastrointestinal inflammatory disorders. It causes issues in about 40% of developed countries’ population and 80% in the developing countries (Alam et al., 2019). Almost 10% of the world’s population is affected by a gastric ulcer, representing about 4 million people each year (Prabhu and Shivani, 2014; Qaiser et al., 2018).

A significant drawback of the extensive use of a common non-steroidal anti-inflammatory drug (Indomethacin) is the development of gastric ulcers (Song et al., 2020b). Multiple studies consider IND-induced gastric ulcers in rats as a standard model to investigate the underlying pathophysiological and pharmacological mechanisms (Liu et al., 2015; Gomaa et al., 2018). Impairment of the release of gastro-protective factors such as cyclooxygenase COX, PGE_2_, bicarbonate, and mucus in addition to increased gastric acid secretion, free radical formation, and excessive generation of inflammatory mediators, e.g., TNF-α, are suggested to be the underlying pathogenic mechanisms of IND-induced gastric ulcers (Abbas and Sakr, 2013).

Available antiulcer drugs, without suspicion, has caused a decline in the incidence of gastric ulcers. Still, their excessive use is associated with several adverse effects like impotence, arrhythmias, hyperplasia, and hematopoietic changes as well as a high chance of recurrence after ending their use (Eraslan et al., 2020). The search for safe, effective, readily accessible, and affordable antiulcer therapy is therefore a medical necessity.

Many phytochemicals derived from plants are used to treat human diseases (Lim et al., 2019). The World Health Organization (WHO) estimated that about 75% of the world’s population, especially in developing countries, trust and rely on herbal medicines for the prevention and treatment of multiple ailments. About 10% of the population relies on herbal medicine for the treatment or prevention of digestive disturbances (Antwi et al., 2009; Aziz, 2013).

Pomegranate trees are widely grown in Egypt and Mediterranean areas (Sharifiyan et al., 2016). It is not only cultivated for its edible fruits, but since prehistoric times, various parts of the plant including its bark, leaves, rinds, seeds, and flowers have been processed for use in disease control (Jayaprakasha et al., 2006). In Egyptian beliefs, the dried pomegranate pulp and husk are widely used to treat abdominal colic, ulcerative colitis, intestinal worms, piles, cough, infertility, and inflammatory skin conditions (Labib and El-Ahmady, 2015). All of its parts are rich in many forms of polyphenols such as flavonoids, anthocyanins, and hydrolysable tannins that exert multiple therapeutic benefits due to their antioxidant, anti-diarrheal, anti-inflammatory, anti-cancer, antimicrobial, and wound healing activities (Gubitosa et al., 2018).

Recently, many health workers have highlighted the gastric ulcer protective and healing effects of pomegranate peel and seeds against NSAIDs-induced gastric ulcers in rats (Guerrero-Solano et al., 2020). Unfortunately, the ability of the phenolic components of pomegranate to be absorbed in the GIT is not great as a result of a weak binding with the mucosa and its low solubility in water; this decreases their bioavailability (Kawser Hossain et al., 2016).

Technological advances in nanomedicine have led to the discovery of several pioneering carrier mechanisms for bioactive phytochemicals. Oral polymeric nanoparticles are considered to be new transporters of medications owing to their nanoscopic size, bio-adhesion, targetability, and ensured perfect adhesion to the mucosa with the sustained release of medicine in the GIT, therefore bypassing the gastrointestinal digestive enzymes and boosting their bioavailability (Anter et al., 2019). Binding herbal extracts with nanoparticles therefore increase their intestinal absorption.

Chitosan is a naturally occurring polysaccharide molecule capable of splitting into multiple monomer molecules both *in vivo* and *in vitro* interacting with living cells with no side effects (Patel et al., 2013). Therefore, in the field of nanotechnology, chitosan and its derivatives are selected solely due to their characteristics of being non-toxic, biodegradable, inert, and because of their immune-enhancing properties (Ye et al., 2013).

To our knowledge, there is currently no literature considering the administration of pomegranate as a nanoparticle for the management of gastric ulcers.

We therefore designed our current study to investigate the possible effects of pomegranate loaded nanoparticles (PLN) on the IND-induced gastric ulcer model in adult rats *via* exploring its impact on inflammatory, oxidative stress markers as well as cytoprotective mucosal PGE_2_ and NO levels and comparing the results with traditional antiulcer agent pomegranate peel extract (PPE). Furthermore, histological and immunohistochemical examination of gastric mucosa were also done.

## Materials and Methods

### Drugs and Chemicals

Indomethacin was purchased from Kahira Pharm. & Chem. Ind. Co., (Cairo, Egypt). Low molecular weight chitosan (CS) and sodium tripolyphosphate (TPP) were obtained from Sigma Chemical Co., (St. Louis, MO, United States). All other chemicals and reagents were of analytical grade and were obtained from standard commercial suppliers.

### Plant Extraction and Identification of Doses

#### Preparation of Pomegranate Peel Extract

Fresh pomegranate fruits (3 Kg), *Punica granatum* L. family Punicaceae (Assiuty cultivar) were obtained from the local market of Assiut governorate, Egypt, in September 2019. The fruits were thoroughly washed with distilled water, peeled, and the collected peels were air-dried. Dried peels (250 g) were ground and macerated in 1 L methanol: distilled water mixture [7:3 v/v] for 48 h, then sonicated for 30 min at ambient temperature. The mixture was filtered, and this step was repeated three times to ensure full extraction of the peels. The combined hydro-alcoholic extract was evaporated to dryness under reduced pressure at (45°C) until all the solvent was removed; to yield (50 g) dried pomegranate peel extract (Venkataramanamma et al., 2016).

#### Preparation of Pomegranates Loaded Nanoparticles (PLN)

Pomegranate loaded nanoparticles (PLN) were prepared by ionic gelation method at different CS/TPP weight ratio. Briefly, different concentrations of chitosan solutions were prepared in 1% w/v glacial acetic acid and filtered. Sodium tripolyphosphate solution (TPP) (0.2% w/v) was prepared in deionized water. Pomegranate peel extract was added to chitosan solution under constant stirring to obtain different formulations of pomegranate-loaded chitosan nanoparticles. The mixture was sonicated for 5 min, and TPP solution will be added in a dropped manner with continuous stirring (Guan et al., 2011).

The morphological characteristics of PLN (1 mg/ml at CS: TPP ratio of 6:1) were investigated by transmission electron microscopy (TEM, Model 100 CX II, Tokyo, Japan). The nanoparticles were placed onto copper grids and dried overnight at room temperature (Katas et al., 2013). They were stored at 4°C for 30 days. The nanoparticle size, polydispersity index (PDI), and Zeta-potential were measured using Malvern Autosizer 4800 equipped with a 488-nm Uniphase argon-ion laser (Malvern Instruments, Worcestershire, United Kingdom) to study the effect of storage on the stability of the nanoparticles. All these measurements were performed in triplicate at a scattering angle of 173° and a temperature of 25°C.

The encapsulation efficiency of PLN was determined *via* centrifugation of the aqueous solution at 14,000 rpm for 60 min. After that, the amount of free pomegranate was measured at a wavelength of 265 nm using a UV-Vis spectrophotometer (Thermo Scientific, Waltham, MA, United States) (Guan et al., 2011). The pomegranate content (%) (Encapsulation efficiency) was calculated using the following equation = (amount of the drug in the nanoparticles/amount of the drug initially added) × 100.

### Rats Maintenance and Ethical Considerations

Twenty-four adult male Wister albino rats, weighing (180–220 g), were bred in clean, spacious cages (up to four per cage) at the animal house of the College of Medicine, Assiut University, Assiut, Egypt. An aerated room was prepared for maintaining rats under standard circumstances of light and temperature (25 ± 5°C). They were fed standard rat chow and tap water was accessible *ad libitum*. The animals were habituated for handling and testing environments for 4 days before the commencement of experiments. All efforts were made to prevent harming the animals throughout the experimental protocol and the collection of samples. All experimental procedures were done following the standards outlined in the guidelines for the care and use of experimental animals by the committee for supervision of experiments on animals and were approved by the ethics committee at the College of Medicine, Assiut University, Assiut, Egypt (IRP number 17300195).

### Induction of Gastric Ulcer

Concisely, prior to ulcer induction, rats were kept fasted with free access to water for 24 h. After that, rats received a single oral dose of IND (50 mg/kg) by gastric gavage. Acute ulcers were detected within about 6 h (Liu et al., 2015).

### Experimental Design

Rats were arbitrarily divided into four groups (six rats in each group). (1) The control group: This group received distilled water only. (2) IND-treated group: rats received IND as described above. (3) PLN-treated group: the animals were given IND followed by PLN in a dose of 400 mg/kg/day *via* gastric gavage for 2 weeks (El-Azab et al., 2018). (4) PPE-treated group: rats in this group received IND followed by PPE in the same dose and duration as PLN.

### Collection of Samples and the Gross Picture of Stomach Mucosa

Just before the rats were euthanized, blood samples were collected from the retro-orbital vein of each rat, and then centrifuged at 1008 rpm for 15 min. The transparent sera were stored until use at −20°C. Afterward, rats from all groups were sacrificed through cervical dislocation after chloroform inhalation anesthesia and their stomachs were extracted, opened along the greater curvature, and softly washed with saline to remove all gastric contents and any blood clots. The stomach was placed on a piece of filter paper with the mucosal surface directed upwards and was photographed with a digital camera. The scope of gastric lesions (ulcer index, UI) was evaluated macroscopically by an impartial participant, unaware of the experimental protocol.

The stomach of each rat was divided into two halves. One half was used for histopathological analysis, and the other was immediately preserved in liquid nitrogen and then relocated and stored at −80°C until its use for biochemical and molecular analysis.

### Measurement of Ulcer Index and Percentage of Ulcer Inhibition

The calculation of the ulcer score for each group was done as the mean number of ulcers in the stomach of each rat in each group (Table 1). Then, the ulcer score of each rat was multiplied by 100 to obtain an ulcer index (UI). Subsequently, calculation of the percentage of ulcer inhibition (inhibition index) was measured as 100% × (UI of the ulcer group – UI of the treated group)/UI of the ulcer group (Boushra et al., 2019).

**TABLE 1:** Ulcer scores and descriptive features.

**Score**	**Features**
0	No ulcer
1	Pinpoint ulcer and changes limited to superficial layers of mucosa
2	Ulcer less than 1 mm in size
3	Ulcer more than 1 mm but less than 2 mm in size
4	Ulcer more than 2 mm or perforated ulcer

### Biochemical Measurements

#### Estimation of the Serum Level of Oxidative Stress Markers (TAC and MDA)

Total antioxidant capacity (TAC) in serum was determined by colorimetric method using a trade available kit (Cat. no# TA 2513) obtained from Bio-Diagnostics Company, Egypt, following previously outlined procedures (Koracevic et al., 2001). The anti-oxidative capacity in rat serum is measured by the reaction of antioxidants in the sample with a known amount of exogenously supplied hydrogen peroxide (H_2_O_2_), resulting in removing a specific amount of H_2_O_2_. The remaining H_2_O_2_ is estimated colorimetrically by an enzymatic reaction, which involves the conversion of 3, 5, dichloro–2–hydroxyl benzensulphonate to a colored product. The level of serum malondialdehyde (MDA), a major lipid peroxidation product, is measured colorimetrically using a commercially available kit (Cat. no# MD2529) obtained from Bio-Diagnostics Company, Egypt, following previously outlined instructions (Ohkawa et al., 1979). This assay is based on the reaction of thiobarbituric acid (TBA) with MDA in an acidic medium at a temperature of 95°C for 30 min to form a thiobarbituric acid reactive product with a pink color, with its absorbance measured at 534 nm. Serum TAC is measured in mM/L, while serum MDA is measured in nmol/ml.

#### Measurement of the Serum Level of Inflammatory Cytokines (IL-2, IL-6, IL-10)

Serum levels of IL-2, IL-6, and IL-10 expressed in pg/ml were measured by a rat enzyme-linked immunosorbent assay (ELISA) kit (Cat # BMS634, BMS625, and BMS629; respectively) purchased from Thermo Fisher Scientific Co., (Waltham, MA, United States).

#### Measurements of Gastric Protective Factors (PGE_2__)_ and NO in the Stomach Homogenate

The gastric wall was sliced into multiple small pieces that were homogenized by mixing with ice-cold phosphate buffer solution to give 10% homogenate. Then, centrifugation was done at 3000 rpm, the clear non-hemolyzed supernatant was preserved at −20°C to be used for estimation of PGE_2_ and NO. Prostaglandin E_2_ was measured in pg/mg according to manufacturer instructions using a rat PGE_2_ Elisa kit (Cat # CSB-E07967r) purchased from Cusabio Biotech Co., Ltd., (Wuhan, China).

The nitric oxide content (nmol/g) was assessed using the colorimetric Griess approach, supplied by Invitrogen, Thermo Fisher Scientific (Cat no # EMSNO) which depends on calculating its nitrite, a metabolic stable indicator of original NO in agreement with (Miranda et al., 2001). The level of NO is measured by the determination of both nitrate and nitrite concentrations in the serum. The kit uses the enzyme nitrate reductase to convert nitrate to nitrite. Nitrite is then detected as a colored deep purple azo dye product of the Griess reaction that absorbs visible light at 540 nm. Hence, the total nitrite/nitrate is considered as an indirect marker of NO production. The protein content in gastric homogenate samples was measured using a biuret reagent (Gornall et al., 1949).

### Quantitative Real Time-Polymerase Chain Reaction (q-PCR)

Total RNA was extracted from 1 gm gastric mucosal tissue collected in Ribozol solution using Thermo Scientific Gene JET RNA Purification Kit (CAT# K0731), according to the manufacturer’s instructions. The extracted RNA concentration was then measured at 260 nm and 280 nm using NanoDrop^®^ (Epoch Microplate Spectrophotometer, Biotek, VA, United States), in the Medical Research Center, Assiut University, Assiut, and stored at −80°C until further analysis. Complementary DNA (cDNA) was synthesized from RNA using the Thermo Scientific Revert Aid First Strand cDNA Synthesis Kit (CAT# K1622) in a reaction catalyzed by reverse transcriptase using oligo-(dT)-primers. The concentration and purity of the resultant cDNA were measured by NanoDrop before the progression of the protocol. The produced cDNA was diluted 1:5 by PCR grade water and then, stored at −20°C until the subsequent step. Quantitative polymerase chain reaction (qPCR) was performed using the Maxima SYBR Green qPCR master mix (2×) kit (Thermo Scientific Catalog. NO #K0251). After that, the Applied Biosystems 7500 Fast Real-time PCR machine (Applied Biosystems, Germany) was conducted using the resultant cDNA that was amplified in a 25 μl reaction volume PCR using a specific primer for eNOS and titrating the results with a house keeping b-actin gene as an internal control. The PCR cycling conditions were as follows: the initial denaturation step at 95°C for 10 min, followed by 35 cycles of 95°C for 15 s and 60°C for 25 s, and dissociation at 72°C for 30 s and 72°C for 5 min. The primer sequences of eNOS and β-actin genes were described by Saha et al. (2016) and are shown in Table 2. The relative expression level of the eNOS gene versus the β-actin gene was calculated using the 2-ΔΔCt method as described by Navarro et al. (2005), and was represented as the fold change relative to the control group.

**TABLE 2:** Primer sequences of rat eNos mRNA and reference gene rat b actin mRNA.

Rat eNOS Sense	5′ TAC TTG AGG ATG TGG CTG
Rat eNOS Antisense	3′ GTC TTC TTC CTG GTG ATG
Rat b actin sense	5′ TTG TAA CCA ACT GGG ACG ATA TGG
Rat b actin antisense	3′ GAT CTT GAT CTT CAT GGT GCTAGG

### Assessment of Histopathological Alterations

#### Light Microscopic Examination

Specimens from the upper portion of the fundus were excised, fixed in 10% formalin buffered with phosphate for 24 h at 4°C, then passed through ascending grades of alcohol, followed by clearing with xylol and impregnation in pure soft paraffin for 2 h at 50°C which was then embedded in hard paraffin. Paraffin sections (5-μm-thick) were prepared and stained with hematoxylin and eosin (H and E) to verify histological details.

#### Immunohistochemical Study

Tumor necrosis factor α and endothelial nitric oxide synthase in the cells of the gastric fundus mucosa were identified by immunohistochemistry using mouse anti-TNF-α monoclonal antibody (1:300) and a polyclonal antibody against eNOS (1:200) (Santa Cruz Biotechnology, Santa Cruz, CA, United States) as primary antibodies. An immunohistochemical study was conducted using the avidin-biotin peroxidase technique. The specimens were put on positively charged glass slides, underwent complete deparaffinization in xylene, followed by rehydration in descending grades of alcohol. Afterward, the endogenous hydrogen peroxidase activity was inhibited followed by exposure to microwave radiation at 500 W for 10 min in a 10 mM citrate buffer, pH 6.0, to retrieve the antigens. Sections with primary antibodies against TNF-α and e-NOS were then incubated overnight (4°C), using diaminobenzidine (DAB) (Dakopatts, Glostrup, Denmark) as a chromogen. Finally, the slides were rinsed with distilled water and counterstained with hematoxylin. The positive cellular reaction appeared as a cytoplasmic brown color (Ma and Wallace, 2000). Positive TNF-α and e-NOS cells were counted blindly by touch count method in five fields of vision under a 400×-microscope, using the Leica Qwin 500 image analysis computer system (Leica Microsystems Ltd., Cambridge, United Kingdom) at the Histology and cell biology department, Faculty of Medicine, Assiut University, Egypt.

#### Histomorphometric Study

Histomorphometric evaluation of gastric mucosal damage is estimated by measuring the whole thickness of intact mucosa (mucosal thickness) and the thickness of damaged mucosa (lesion thickness) according to a previous study (Natale et al., 2001).

### Statistical Analysis

SPSS program version 21 (SPSS Inc., Chicago, IL, United States) was agreed to be used for the analysis of data. Descriptive statistics in the form of mean ± SD were used for presenting the results of the study. The statistical analysis was carried out using the Kruskal-Wallis *H* test followed by the Mann–Whitney *U* test. Non-parametric Spearman’s correlations were done to estimate the relationships among measured parameters according to Knapp and Miller (1992). The probability value ≤0.05 was regarded statistically significant.

## Results

### Physicochemical Descriptions of the Pomegranate Loaded Nanoparticles

**FIGURE 1:**
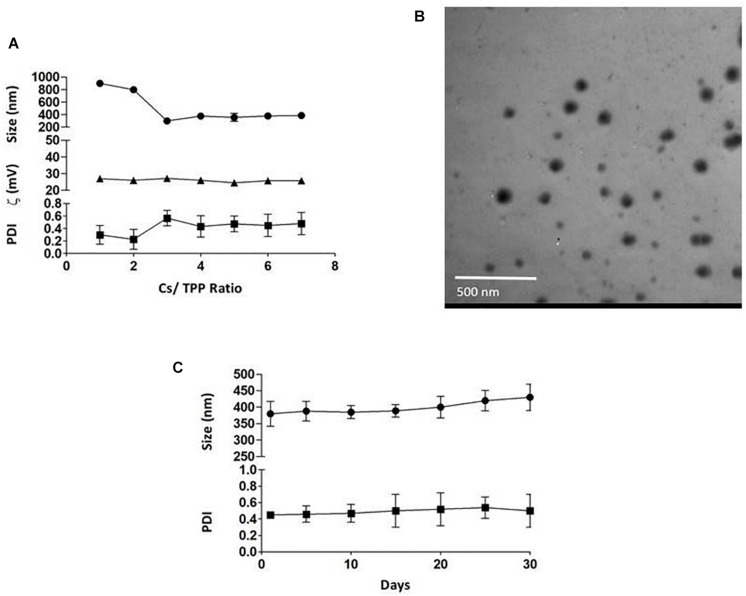
**(A)** the effect of CS/TPP ratio on the size, Zeta potential and PDI of pomegranate extract-loaded CS/TPP nanoparticles. **(B)** TEM image of the pomegranate-loaded nanoparticles (CS: TPP weight ratio of 6:1) without staining at magnifications of 48,000×, scale bar = 500 nm. **(C)** the effect of storage on the size and PDI of pomegranate-loaded nanoparticles at 4°C for 30 days. The values are presented as mean ± SD.

The average particle size of the prepared nanoparticles (1:1 to 7:1 CS/TPP ratio) ranged from 299 to 900 nm, and the PDI ranged from 0.2 to 0.5. The nanoparticles had positive zeta potential ranging from 25 to 27 mV at 25°C (Figure 1A). The shape of PLN was close to sphericity as detected using TEM (Figure 1B). The encapsulation efficiency of pomegranate into the nanoparticles ranged from 80 to 85% (Table 3). The selected nanoparticles were observed at a ratio of 6:1 CS: TPP. These nanoparticles have an optimum size, PDI, and high encapsulation efficiency. The optimized nanoparticles were selected for further characterization. The result showed that the pomegranate had a little increase in size and PDI over the 30 day incubation period. The size and PDI ranged from 380 to 430 nm and 0.45–0.5, respectively, over the 30 day incubation period for PLN (Figure 1C).

**TABLE 3:** Encapsulation efficiency of PLN (pomegranate extract-loaded CS/TPP nanoparticles prepared at a ratio of 1:2 to 6:1 CS/TPP).

**Cs/Tpp Ratio**	**Encapsulation Efficiency (%)**
1	75
2	80.9
3	82.93
4	83
5	81.8
6	85
7	84

*Data were shown as mean ± SD (*n* = 3).*

### Effects of PLN and PPE on Stomach Mucosa Gross Picture, Ulcer Index, and Inhibition Index

**FIGURE 2:**
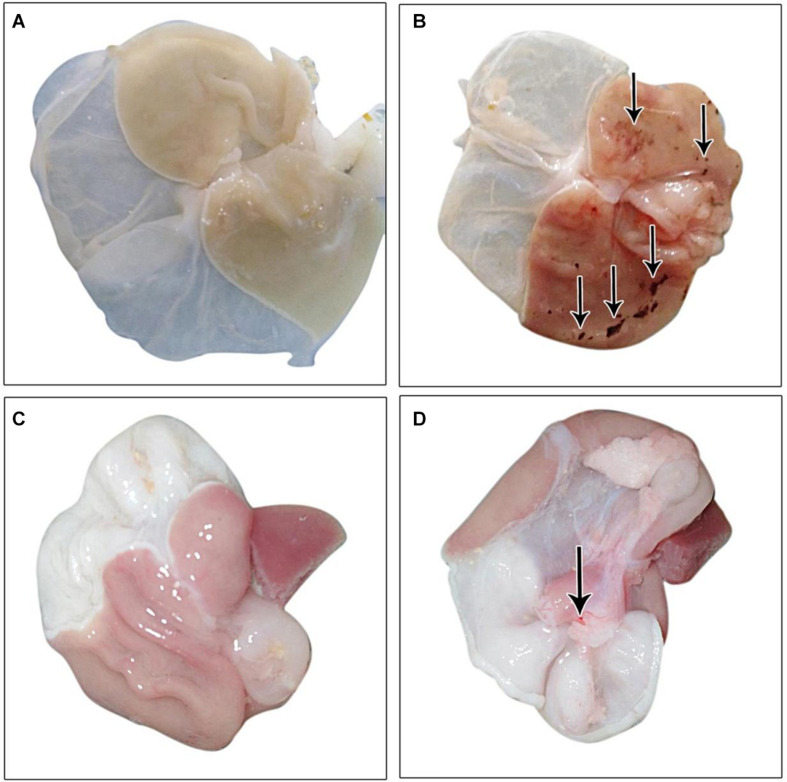
Gross picture of gastric mucosa in all examined groups: **(A)** control group, **(B)** IND-treated group, **(C)** PLN-treated group, **(D)** PPE-treated group.

The gastric mucosa appeared normal with no apparent lesions visible to the naked eye, during the examination in the control group. Conversely, an inspection of the gastric mucosa of IND-treated rats revealed congested mucosa with the appearance of multiple ulcers that achieved an ulcer score of 5.667 ± 1.64 mm and a group UI of 566.7. Treated rats with PLN and PPE markedly decreased these lesions, as proven by significant decreases in ulcer scores versus the IND-treated group. Rats in the PLN-treated group had a mean score of 0.1667 ± 0.4082 mm, while those in the PPE-treated group had a value of 1.333 ± 0.5164 mm. After calculating the percentage of ulcer inhibition, PLN-treated rats showed a group UI of 16.67 and a net inhibition index of 97.06%. To a nearly similar extent, the PPE-treated animals had a group UI of 133.3 and a net inhibition index of 76.48%, denoting a higher gastroprotective effect of PLN (Table 4 and Figure 2).

**TABLE 4:** shows Mean ± SD of ulcer scores, indices and the net inhibition indices.

**Groups**	**Control**	**IND-treated**	**PLN-treated**	**PPE-treated**
Ulcer score	–	5.667 ± 1.64	0.1667 ± 0.4082	1.333 ± 0.5164
Ulcer index	–	566.7^a^	16.67^b,c^	133.3^a,b^
Net inhibition index	–	0	97.06%	76.48%

*^*a*^P: significant vs. the control group. ^*b*^P: significant vs. IND-treated group; ^*c*^P: significant vs. PPE-treated group (*p* ≤ 0.05).*

### Effects of PLN and PPE on the Serum Levels of Oxidative Stress Markers (MDA and TAC)

The level of the major lipid peroxidation product (MDA) was elevated significantly in the serum of adult male rats given IND as compared to the control group (*p* = 0.041). On the contrary, a significant decrease in the serum level of TAC was found in the IND-treated group versus the control group (*p* = 0.004). Hence, this proved the occurrence of a state of mal-equilibrium among oxidants and antioxidants in IND-treated rats (Table 5). Oral intake of PLN restored the normal balance between oxidants and antioxidants as evidenced by a significant reduction in the serum level of MDA (*p* = 0.002) and a marked increase in the serum level of TAC in comparison with the IND-treated group (*p* = 0.002). Moreover, the serum level of MDA was insignificantly reduced in rats treated with PPE versus the control and IND-treated rats. Furthermore, TAC level in serum was elevated to a significant extent versus the IND-treated group (*p* = 0.002) with no significant difference from the control rats. Astonishingly, these effects are more pronounced in rats treated with PLN, indicated by a significant diminution in the serum level of MDA compared to the control and PPE-treated groups (*p* = 0.004 and 0.004; respectively) (Table 5).

**TABLE 5:** Serum level of malondialdehyde (nmol/mL) and TAC (mM/L) in the different studied groups.

**Groups**	**Control**	**IND-treated**	**PLN-treated**	**PPE-treated**
MDA	8.98 ± 1.27	12.8 ± 3.12^a^	6.22 ± 1.09^a,b,c^	9.22 ± 1.23
TAC	1.33 ± 0.31	0.63 ± 0.27^a^	1.97 ± 0.54^b^	1.3 ± 0.14^b^

*Data are mean ± SD (*n* = 6 for each group). ^*a*^P: significant vs. the control group. ^*b*^P: significant vs. IND-treated group; ^*c*^P: significant vs. PPE-treated group (*p* ≤ 0.05).*

### Effects of PLN and PPE on the Serum Levels of Pro-inflammatory Cytokines (IL-2, IL-6, and IL-10)

**FIGURE 3:**
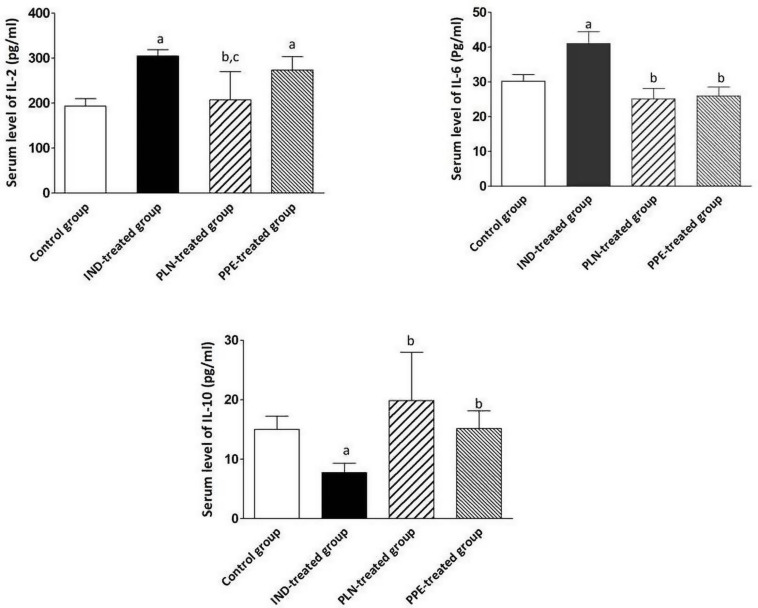
Serum levels of inflammatory cytokine status (IL-2, IL-6, IL-10) in pg/ml in the different studied groups. Data are mean ± SD (*n* = 6 for each group). ^a^P: significant vs. the control group. ^b^P: significant vs. IND-treated group; ^c^P: significant vs. PPE-treated group (*p* ≤ 0.05).

Regarding the inflammatory cytokines, the serum level of IL2 and IL6 raised significantly in rats given single oral IND in comparison with the control group (*p* = 0.002, 0.026; respectively). Administration of PPE reduced the serum level of IL2 with no significant difference to the IND-treated group, but the level is still significantly higher than the control group (*p* = 0.002). Surprisingly, PLN decreased the serum level of IL2 to a marked degree compared to the IND-treated and PPE-treated groups (*p* = 0.002 and 0.041; respectively) and normalized its level with no significant difference from the control group. Moreover, intake of PLN and PPE reduced the serum level of IL6 to a significant extent as compared to the IND-treated group (*p* = 0.009 and 0.009; respectively) (Figure 3). On the other hand, it was found that the serum level of the anti-inflammatory cytokine (IL-10) declined markedly in rats given IND versus the control, PPE-treated, and PLN-treated rats (*p* = 0.002, 0.002, and 0.004; respectively). It is evident that administration of pomegranate in both forms achieved normalization of the IL-10 serum level with no significant differences from the control rats (Figure 3).

### Effects of PLN and PPE on the Gastric Protective Factors (PGE_2_ and NO)

**FIGURE 4:**
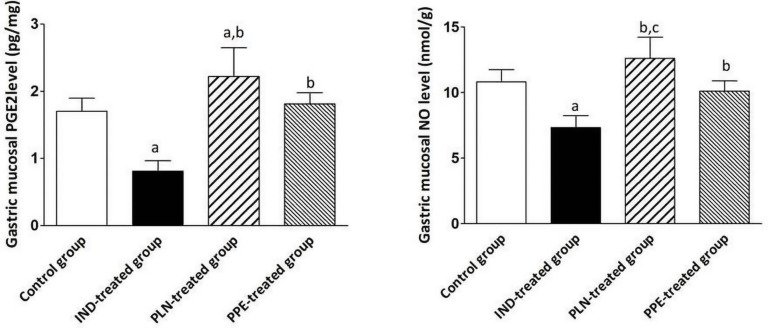
Stomach homogenate levels of PGE_2_ (pg/mg) and NO (nmol/g) in all examined groups. Data are mean ± SD (*n* = 6 for each group). ^a^significant compared to the control group. ^b^significant compared to IND-treated group; ^c^significant compared to PPE-treated group (*p* ≤ 0.05).

Considering the gastric tissue level of PGE_2_, it was reduced to a significant degree in the IND-treated group compared to the control group (*p* = 0.002). Furthermore, oral administration of PLN and PPE raised the level of PGE_2_ to a marked degree in comparison with the IND-treated group (*p* = 0.002 and *p* = 0.002, respectively). Although PPE oral administration insignificantly increased the level of PGE_2_, but PLN succeeded in elevating the level of PGE_2_ to a marked degree compared to the control group (*p* = 0.041) (Figure 4). Similarly, it was found that the stomach tissue level of NO was markedly raised in the control, PLN, and PPE-treated groups versus the IND-treated groups (*p* = 0.002, 0.002, and 0.002; respectively). This increase is more prominent in rats treated with PLN than the PPE-treated group (*p* = 0.015) (Figure 4).

### Effects of PLN and PPE on Expression of eNOS in Gastric Tissue

**FIGURE 5:**
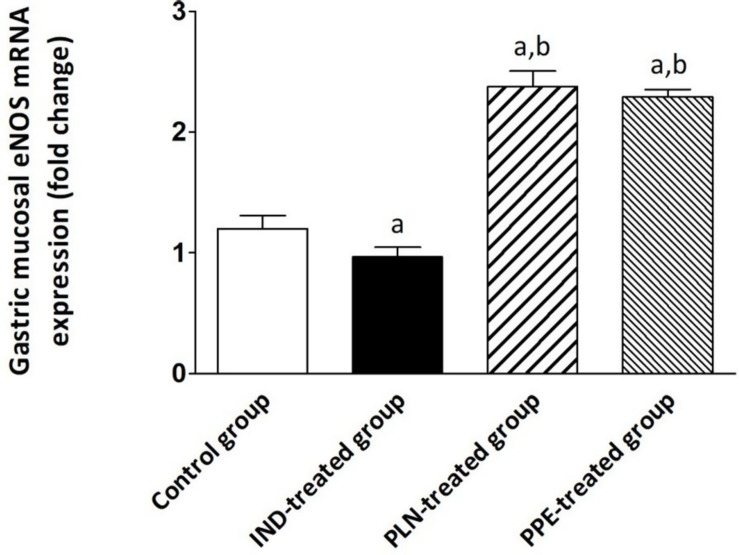
The expression of the gastric eNOS mRNA in the different experimental groups. Mean ± SD (*n* = 6) were shown. The relative expression of the eNOS gene versus a housekeeping reference gene (β-actin) was calculated using the equation of 2^–ΔΔCt^ and expressed fold change relative to the control group, IND-treated group, PPE-treated group. ^a^significant as compared to the control group (*P* ≤ 0.05)., ^b^significant as compared to IND-treated group (*P* ≤ 0.05).

The gastric tissue expression of eNOS was attenuated by about 0.97-fold in IND-treated rats, reaching a significant extent in comparison with the control, PLN-treated, and PPE-treated groups (*p* = 0.004, 0.002, and 0.002; respectively). Amazingly, a marked upregulation of eNOS expression was noticed after treating rats with oral PLN (2.38) or PPE (2.29), with a statistically significant degree versus the control animal group (*p* = 0.002 and *p* = 0.002; respectively) (Figure 5).

### Correlation

**FIGURE 6:**
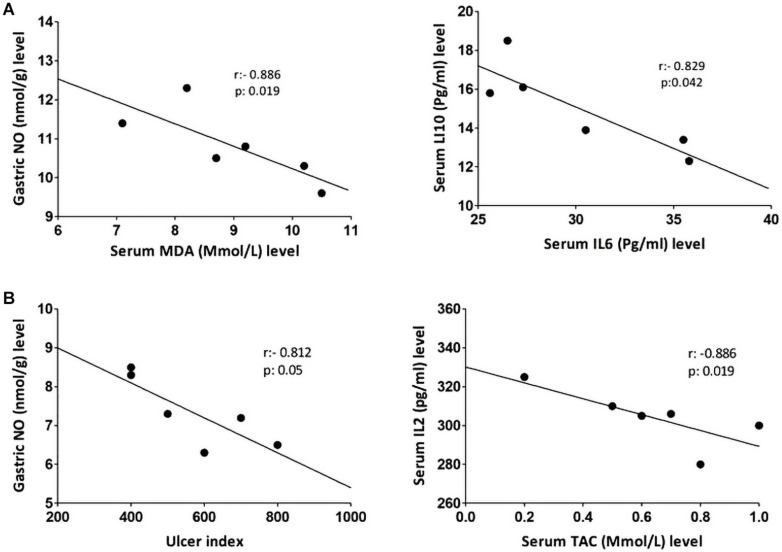
Linear regression and correlations between serum oxidative stress, inflammatory cytokines, gastric NO levels, and ulcer index in the control **(A)** and IND-treated **(B)** groups.

**FIGURE 7:**
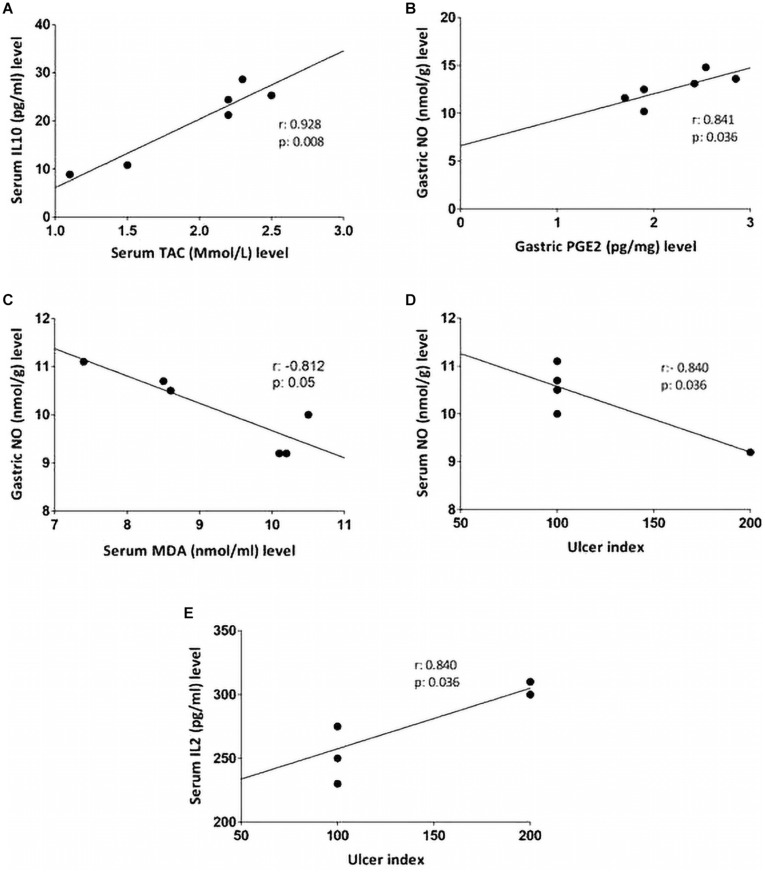
Linear regression and correlations between serum oxidative stress, inflammatory cytokines, gastric protective factors (PGE2, NO), and ulcer index in PLN-treated **(A,B)** and PPE-treated groups **(C–E)**.

Non-parametric spearman’s correlation was performed individually in each group (control, IND-treated, PLN-treated, and PPE-treated). Gastric NO level had significant negative correlations with serum MDA in the control group and with ulcer index in the IND-treated group. Moreover, marked negative correlations were computed between IL6, IL10 in the control, and between IL2, TAC in IND-treated groups (Figure 6). On the contrary, oral administration of PLN resulted in significant positive correlations between both gastric protective factors (NO, PGE_2_) and serum TAC, IL10, whereas no significant correlations were found among ulcer index and all estimated biochemical parameters. However, in the PPE-treated group, ulcer index significantly negatively correlated with gastric NO level and positively with serum IL2 level. Moreover, serum MDA showed marked negative correlations with gastric NO level (Figure 7).

### Light Microscopic Examination and Histomorphometric Study

**FIGURE 8:**
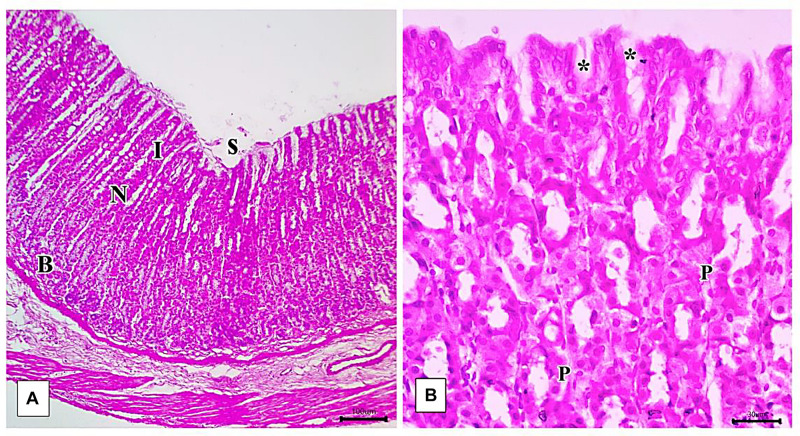
Photomicrographs of the fundic mucosa of the control group illustrating **(A)** normal histologic structure of surface lining epithelium (s), longitudinally arranged healthy-looking glands consisting of the isthmus (I), neck (N), body, and base (B) (H&E × 100, scale bar = 100 μm). **(B)** the upper part of the fundic gland surface epithelial columnar mucous secreting cells (s) short gastric pits (*). Notice that parietal cells are distinguished by their rounded nuclei and acidophilic cytoplasm (P) (H&E × 400, scale bar = 30 μm).

**FIGURE 9:**
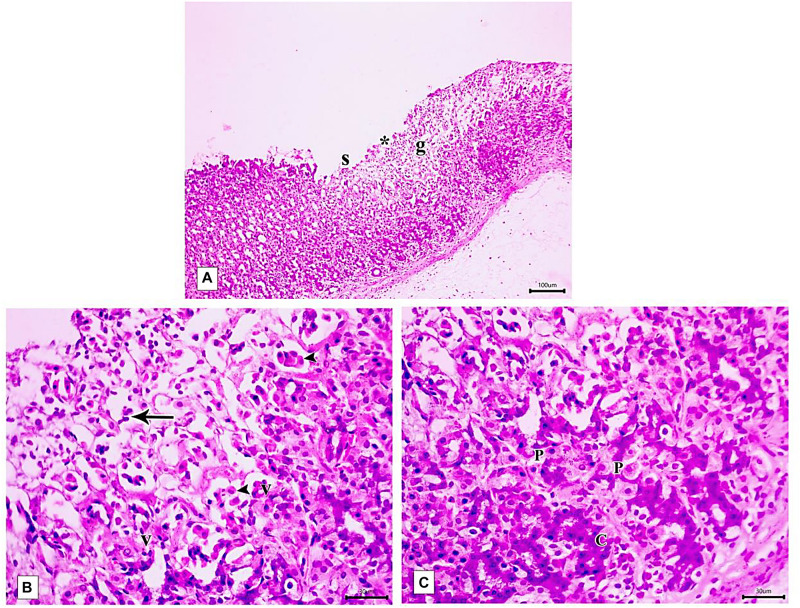
Photomicrographs of the fundic mucosa of IND-treated group showing **(A)** loss of surface epithelial layer(s) and gastric pits (*), reduced mucosal thickness and distorted arrangement of glands in the mucosa (g) (H&E × 100, scale bar = 100 μm). **(B)** heavy leucocyte infiltration of the lamina propria (arrows), vacuolization of some cells (v) with the detachment of epithelial cells into the glandular lumen (arrowhead) (H&E × 400, scale bar = 30 μm). **(C)** the base of fundic gland showing areas of deeply stained nuclei and cytoplasm of chief cells (C), marked proliferation of parietal cells (P) (H&E × 400, scale bar = 30 μm).

Light microscopic examination of the fundic mucosa stained with H&E of the control group showed intact surface epithelium, consisting of simple columnar mucous secreting cells that extend into the lamina propria forming pits (Figure 8A). Fundic glands run perpendicular to the surface, occupying the whole thickness of the lamina propria (Figure 8B). The mucosal injury was observed in IND-treated rats in the form of patchy areas of erosions and ulcerations. The surface epithelium is damaged with the detachment of cells and lost gastric pits (Figure 9A). The glandular architecture is distorted. Leucocyte infiltration of the lamina propria and exfoliated dead cells appeared in the lumen of the upper half of the glands (Figure 9B). At the base of the fundic gland, deeply stained chief cells and predominance of parietal cells were observed (Figure 9C).

**FIGURE 10:**
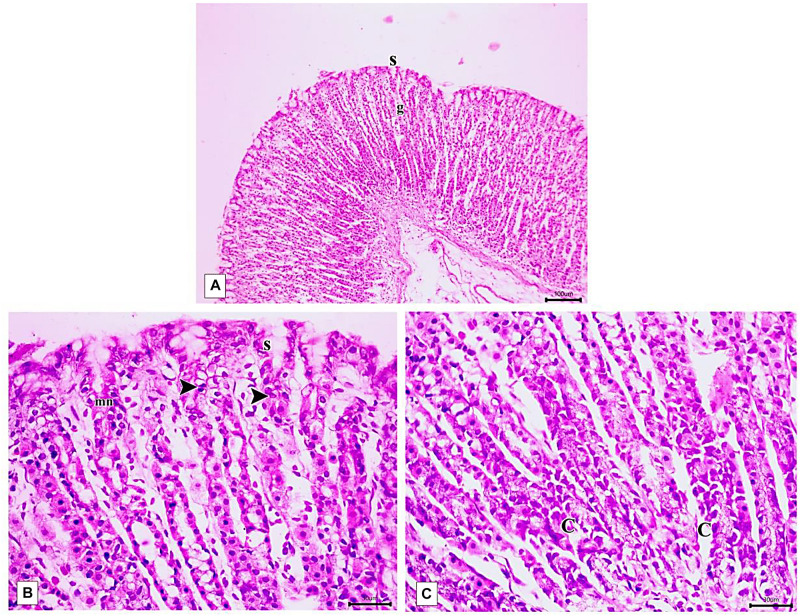
Photomicrographs of the fundic mucosa of PLN-treated group showing **(A)** intact surface epithelial layer(s) with maintained glandular architecture (g) (H&E × 100, scale bar 100 μm). **(B)** mitotic figures in the region of the neck of the gland (arrowhead), reappearance of surface mucous secreting cells (s) mucous neck cells(mn). (H&E × 400, scale bar = 30 μm). **(C)** the basal part of fundic glands showing predominant chief cells having secretory granules (C), cells with vacuolated cytoplasm near the base (H&E × 400, scale bar 30 μm).

**FIGURE 11:**
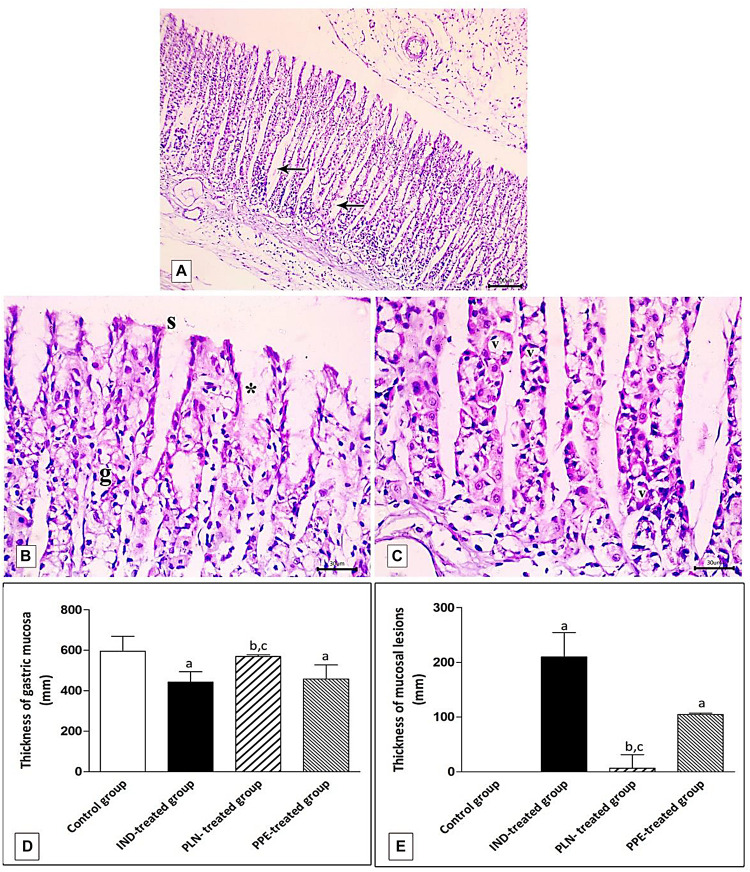
Photomicrographs of the fundic mucosa of PPE- treated group showing **(A)** noticeable disappearance of gastric lesions compared to IND-treated group. Wide Spaces appear in the lamina propria between glands (arrow) (H&E × 100, scale bar = 100 μm). **(B)** attenuation of surface epithelium(s) with denudation of the gastric pits (*). The fundic glands show partial incomplete restoration of their lining epithelium (g) (H&E × 400, scale bar = 30 μm). **(C)** the basal part of fundic glands showing vacuolation of chief cells (v) with deeply stained nuclei (H&E × 400, scale bar = 30 μm). **(D)** quantitative analysis of the mucosal thickness in the stomach fundus of the studied groups. **(E)** quantitative analysis of lesion thickness in the stomach fundus of the studied groups. Data are presented as mean ± SD. Kruskal-Wallis *H* test followed by the Mann-Whitney *U* tests were used for analyzing the results. ^a^significant as compared to the control group (*P* ≤ 0.05). ^b^significant as compared to IND-treated group (*P* ≤ 0.05), ^c^significant as compared to PPE-treated group (*P* ≤ 0.05).

The PLN-treated group showed restoration of the normal integrity of the fundic mucosal layer (Figure 10A) with proliferation of cells in the neck of the fundic glands, reappearance of surface epithelium and gastric pits (Figure 10B). Restoration of glandular architecture occurred with the predominance of chief cells having numerous secretory granules. Still, some areas showed moderate glandular breakage and cells with vacuolated cytoplasm near the base (Figure 10C). However, partial restoration of fundic mucosa and reduced erosions were noticed in rats treated with PPE. Wide spaces appeared in the lamina propria in between the fundic glands (Figure 11A). Attenuation of the surface epithelium with denudation of the gastric pits was observed (Figure 11B). At the gland base, vacuolization of some cells, notably chief cells with deeply stained nuclei were seen (Figure 11C).

Histomorphometric analysis revealed a significant reduction of the thickness of gastric mucosa with a marked increased ulcerative lesion thickness in the IND-treated group as compared to the control group (*p* = 0.000, *p* = 0.002; respectively). On the contrary, PLN elevated the mucosal thickness and markedly reduced lesion thickness compared to the IND-treated group. Hence, it succeeded in restoring their levels to the normal values. On the other hand, treatment with natural PPE insignificantly elevated mucosal thickness as it is still markedly lower than the control group (*p* = 0.000). The lesion thickness decreased insignificantly versus the IND-treated group but showed a higher value than the control group (*p* = 0.001). Noticeably, the improvements in mucosal thickness and decrement of lesion thickness were more prominent in rats administered PLN other than the natural extract (*p* = 0.002 and *p* = 0.004; respectively) (Figures 11D,E).

### Immunohistochemical Identification of TNF-α and eNOS in Gastric Mucosa

**FIGURE 12:**
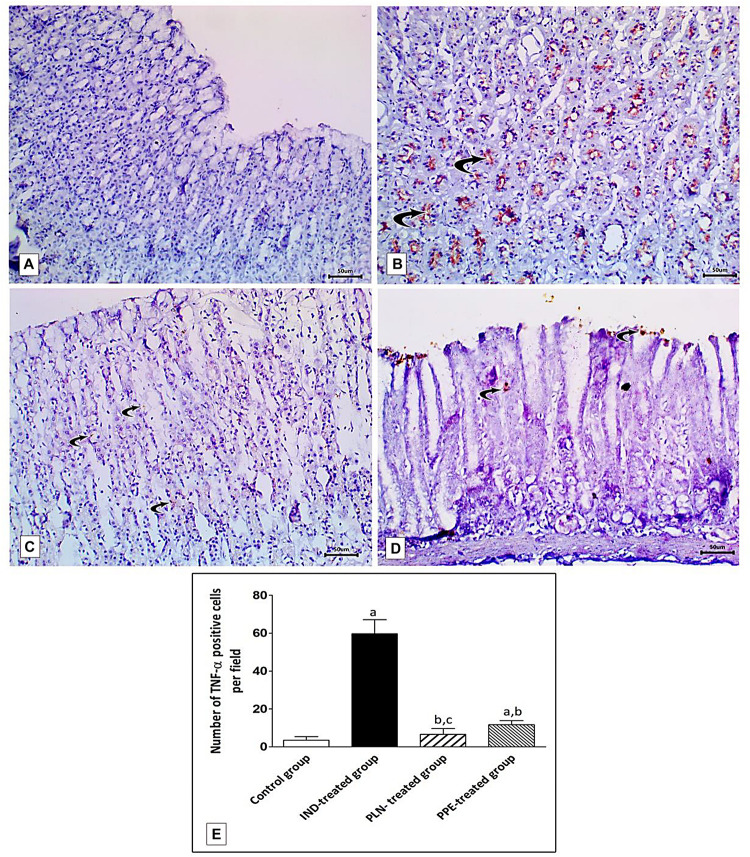
Sections from the fundic mucosa demonstrating immunohistochemical reactivity to TNF-α in the different studied groups. **(A)** the control group shows weak TNF-α immunoreaction in the gastric mucosa. **(B)** IND-treated group shows highly positive TNF-α immunoreaction especially in the basal glands (curved arrow). **(C)** PLN-treated group shows weak positive TNF-α immunoreaction (curved arrow). **(D)** PPE-treated group showing mild positive TNF-α immunoreaction especially in surface epithelium (Immunostaining for TNF-α × 200, scale bar = 50 μm). **(E)** quantitative analysis of the mean count of TNF-α immunopositive cells in the fundic mucosa of the studied groups. Data are presented as mean ± SD. Kruskal-Wallis *H* test followed by the Mann-Whitney *U* tests were used for analyzing the results. ^a^significant as compared to the control group (*p* ≤ 0.05). ^b^significant as compared to IND-treated group (*p* ≤ 0.05), ^c^significant as compared to PPE-treated group (*p* ≤ 0.05).

Examination of rat gastric mucosa for immunohistochemical detection of inflammatory markers (TNF-α) showed weak positive TNF-α expression in the control group (Figure 12A). On the other hand, highly positive brown detection of TNF-α, especially in basal glands, was noticed in the IND-treated group (Figure 12B), and this increased count of immunopositive cells reached a significant level as compared to the control group (*p* = 0.002) (Figure 12E). Oral treatment with PLN and traditional PPE demonstrated weak positive (6.67) and mild positive (11.67) TNF-α immunoreactive cells (Figures 12C,D; respectively), especially in the surface epithelium, and their count reached a significant decrease versus the IND-treated group (*p* = 0.002 and *p* = 0.002; respectively) (Figure 12E). Furthermore, the nanoparticles of pomegranate were able to restore the normal expression of TNF-α in the gastric mucosa with no significant difference to the control group. In contrast, traditional PPE reduced the expression of TNF-α but is still significantly higher as compared to the control group (*p* = 0.002) (Figure 12E).

**FIGURE 13:**
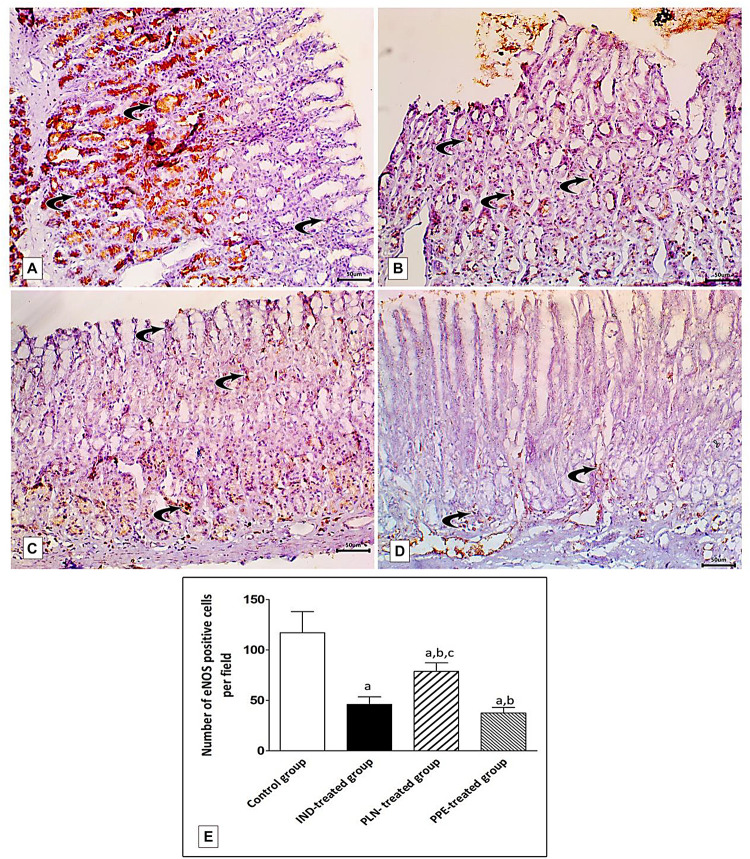
Photomicrographs of immunohistochemical staining of e-NOS in fundic mucosa showing: **(A)** the control group showing highly positive strong expression of e-NOS in glandular epithelial cells and in blood capillaries in the lamina propria (curved arrow). **(B)** IND-treated group showing weak positive expression confined mainly in blood capillaries of the lamina propria (curved arrows). **(C)** PLN-treated group showing moderate positive expression in the vascular endothelium, surface epithelium and glandular epithelial cells (curved arrow). **(D)** PPE-treated group showing weak positive expression more prominently in the vascular endothelium of the lamina propria (curved arrow) (Immunostaining for eNOS × 200, scale bar = 50 μm). **(E)** quantitative analysis of the mean count of e-NOS immunopositive cells in the fundic mucosa of the studied groups. Data are presented as mean ± SD. Kruskal-Wallis *H* test followed by the Mann-Whitney *U* tests were used for analyzing the results. ^a^significant as compared to the control group (*p* ≤ 0.05). ^b^significant as compared to IND-treated group (*p* ≤ 0.05), ^c^significant as compared to PPE-treated group (*p* ≤ 0.05).

Specimens of normal gastric mucosa demonstrated strong immunoreactivity for e-NOS in the glandular epithelial cells and in blood capillaries of the lamina propria (Figure 13A). IND-treated ulcerated tissues showed weak positive expression confined mainly to the endothelium of blood capillaries of the lamina propria (Figure 13B). The mean count of immunopositive cells was significantly decreased as compared to the control, PLN, and the PPE-treated groups (*p* = 0.002, *p* = 0.002, and *p* = 0.041, respectively). On the other hand, oral administration of the nanoparticles showed moderate positive expression in the vascular endothelium, surface epithelium, and glandular epithelial cells (Figure 13C), but still the mean count of e-NOS immunopositive cells was markedly lower than the control group (*p* = 0.002). In an approximately similar manner, the PPE-treated group showed mild positive eNOS immune-positive cells in the vascular endothelium of blood capillaries of the lamina propria (Figure 13D) with a still markedly lower expression than the control group (*p* = 0.002).

Surprisingly, the expression of TNF- α was reduced and that of eNOS was elevated to a marked degree in the PLN-treated rats versus the PPE-treated rats, revealing a rapid and prominent effect of nanoparticles in promoting the healing of a gastric ulcer (*p* = 0.015 and *p* = 0.002; respectively) (Figures 12E, 13E).

## Discussion

Gastric ulcer is one of the most important problems in developing countries that may be attributed to the exposure of stomach mucosa to various harmful factors (Antwi et al., 2009). The detailed etiopathogenesis of this ailment is still unclear (Ramamurthy and Marueen, 2018). The current study searched the effects of PLN against IND-induced gastric ulcers, and compared the effects with traditional PPE. Increased consumption of NSAIDs was believed to be responsible for nearly 25% of gastric ulcer cases (Adhikary et al., 2011). Of them, Indomethacin, an effective medicine used in chronic arthritis, is most frequently cited as a cause of gastric mucosal injury. In addition to causing gastric ulceration, it also delays ulcer healing (Hawkins and Hanks, 2000). This was proved in our study by the appearance of multiple ulcers, a marked increase in the ulcer index, structural alterations in the sections from fundic gastric mucosa that appeared in the form of patchy areas of erosions and ulcerations together with loss of gastric pits, and a noticeable prominent leucocyte infiltration in the lamina propria, confirming the inflammatory triggering role of IND.

Excessive generation of free radicals, inflammatory cytokines, and increased HCL secretion were assumed to be the underlying mechanisms of IND-mediated gastric mucosal injury. The present study revealed a marked increase in the serum levels of MDA, a common metabolite of lipid peroxidation, and decreased TAC; generating a condition of oxidative overload. This was partially in concordance with previous studies (Liu et al., 2015; Al-Quraishy et al., 2017; Gomaa et al., 2018). This may be attributed to IND-mediated suppression of the respiratory chain with the resulting release of mitochondrial cytochrome c into the cytosol and subsequent generation of reactive oxygen species. Thereafter, progressive intracellular ATP depletion, liberation of lysosomal enzymes, hydroxyl anion mediated degeneration of hyaluronic acid, the basic constituent of epithelial cells basement membrane, as well as peroxidation of tissue lipids, proteins, and nucleic acids represent prominent consequences of raised free radical formation culminating in mucosal injury (Matsui et al., 2011). Furthermore, the free radicals enhanced vascular permeability with an enrollment of motivated neutrophils into the gastric mucosa (El-Abhar, 2010) that adhere directly to gastric endothelium, occluding microvasculature and decreasing mucosal blood flow, leading to ulceration (Shim and Kim, 2016).

The probable danger of extensive oxidative stress is its ability to provoke an inflammatory response in gastric mucosa (Sun et al., 2015). The role of inflammatory cytokines in the pathogenesis of gastric ulcers was documented in former studies (El-Ashmawy et al., 2016; Katary and Salahuddin, 2017; Gomaa et al., 2018; Abdelfattah et al., 2019). The marked increase in levels of inflammatory mediators IL2, IL6 in the serum of IND-treated rats, in addition to an intense increase in the count of TNF-alpha gastric mucosal immunopositive cells and diminished serum anti-inflammatory cytokine IL10 levels, found in the present study, proved this hypothesis. A major contributor to IND-induced gastric mucosal injury was shown to be TNF-α (Souza et al., 2004). This pro-inflammatory cytokine, together with oxidants, activate the nuclear factor kappa B (NF-κB) pathway through phosphorylating and suppressing its inhibitor IKB, with subsequent translocation into the nucleus to start transcription of other inflammatory genes that, in turn, induce formation of adhesion molecules on the cell membrane of both neutrophil and endothelial cells facilitating their attraction (Lawrence, 2009; Abdelfattah et al., 2019), thereby accounting for the massive neutrophil and other inflammatory cell infiltration observed in our results. A previous study conducted by Paul (2013) postulated the increased TNF-α expression to be an indicator of gastric inflammation. It is worth noting that infiltrating inflammatory cells could be a major source of reactive oxygen species and subsequent oxidative status (Ganguly and Swarnakar, 2012). Hence, a vicious cycle of inflammation and excessive free radical generation was created in the gastric mucosa overthrowing natural antioxidant defense mechanisms and explaining mucosal injury. These findings were verified by negative correlations between TAC and IL2 in the IND-treated group.

Maintenance of a healthy gastric epithelial mucosal barrier is postulated to depend on adequate gastric PGE_2_ and NO levels. Prostaglandin E_2_ was assumed to increase mucous and bicarbonate ion release, mucosal blood flow, and reduced gastric HCL secretion (Rahim et al., 2014). Nitric oxide in the gastric mucosa works in a paradoxical manner. It is synthesized from L-Arginine in a chemical reaction catalyzed by either cytoprotective constitutive eNOS or by cytotoxic inducible nitric oxide synthase (iNOS) (Abdel-Raheem, 2010). NO generated from eNOS promotes ulcer healing through stimulating vasodilatation, inducing the formation of new blood vessels, quenching free radicals, and ameliorating aggregation of leucocytes; resulting in the restitution of epithelial tissue integrity with the increased mucous release (Nishida et al., 1998; Khattab et al., 2001). Furthermore, NO generated from iNOS, function in gastric ulcer induction *via* the formation of reactive oxygen species and toxic effects on cells (Cho, 2001).

Herein, levels of PGE_2_, NO were markedly attenuated in the gastric ulcer group in compliance with formerly published studies (El-Ashmawy et al., 2016; Allam and El-Gohary, 2017), confirming the ulcerogenic effect of IND *via* increasing HCL secretion. This decrement of gastric PGE_2_ level results in increased gastric acid secretion agreeing with previous reports (Adhikary et al., 2011; Ashraf et al., 2012) where IND was reported to cause alterations in gastric secretions of rats. The predominance of parietal cells in ulcerated regions detected in our histological sections confirm previous findings (Wallace, 2008) where IND significantly augmented acid secretion of parietal cells. At the same time, it did not show any effect on the basal secretion of bicarbonate ions from surface mucous cells. The state of oxidative stress in the gastric mucosa was incriminated in the reduction of PGE_2_ as a result of the inhibition of mucosal cell COX, a rate-limiting enzyme of prostaglandin synthesis, or, may be due to the transformation of PGE_2_ to 8 iso-PGF_2_ alpha, a metabolic end-product of oxidation (Allam and El-Gohary, 2017). The former investigators (Katary and Salahuddin, 2017) clarified the decreased gastric NO content to be due to IND-mediated suppression of gastric eNOS enzyme activity. The genetic and immunohistochemical results of the IND-treated group substantiated this hypothesis where ulcerated tissues showed a significant reduction of eNOS mRNA expression and a decrease in the mean count of eNOS immunopositive cells compared to the control.

Despite the lack of sure scientific evidence supporting the use of herbal extracts in medical fields, the branch of phytotherapy is still considered a supplementary or a complementary prescription for the prevention and treatment of several pathologies (Cravatto et al., 2010). The pomegranate is a highly nutritious fruit, containing many beneficial agents such as polyphenols, alkaloids, tannins and flavonoids, vitamin C, and minerals. These substances possess several therapeutic effects (Elfalleh et al., 2011). Oral intake of either PLN or traditional PPE demonstrated significant reductions of ulcer index, oxidant mediator MDA, inflammatory cytokines IL-2, IL-6, and TNF-α immune-expression in gastric mucosa accompanied with marked elevation of serum levels of TAC, IL-10 denoting alleviation of the stressful conditions of oxidation and inflammation. These results partially agree with previous findings (Chauhan et al., 2017, 2018; Katary and Salahuddin, 2017). The administration of PLN caused about 97.06% inhibition as compared to 76.48% inhibition with ordinary PPE. The gastroprotective effects of punicalagin (found in pomegranate juice) against an ethanol-induced gastric ulcer were confirmed (Katary and Salahuddin, 2017) to be achieved by reducing the protein expression of NF-kB, TNF-α gene expression. A previous study (Rafraf et al., 2017) attributed the anti-inflammatory effect of pomegranate to the downregulation of cytokines such as TNF-α, IL-1, and IL-6. The authors explained that this improvement was due to the polyphenolic contents of the pomegranate, which produce strong antioxidant, anti-inflammatory, and tissue repair properties. The negative correlations between ulcer index and IL2 in the PPE-treated group, and the positive correlation between TAC and IL10 in the PLN-treated group, verified our results.

The basic mechanism for reducing oxidative overload by PPE was *via* upregulation of the cytoplasmic transcription factor Nrf2, by disturbing the Nrf2-Keap1 complex, facilitating the entry of Nrf2 into the nucleus and binding to DNA, enhancing the transcription of the antioxidant genes with consequent increased generation of natural cellular antioxidant enzymes. This, in turn, restored the cellular redox balance, reinforcing the cellular resistance to various harmful agents (Al-Quraishy et al., 2017; Kandeil et al., 2018). A recent study (Patel et al., 2019) explained the antioxidant capacity of PPE to the presence of ellagic acid, where its oxidant scavenging activity was potentiated by anthocyanins phenolic constituents of the extract. Moreover, Hydroxyl and carboxyl groups present in organic compounds of the extract helped them to chelate metal ions and abolish their oxidant damaging effects.

Endothelial nitric oxide synthase exists intracellularly in normal gastric mucosa with distinct distribution patterns showing a greater expression in the corpus and antrum than in the proximal third of the normal human stomach (Rajnakova et al., 1997). The macrophages, endothelial cells, and neural elements in the stomach wall also showed eNOS immunoreactivity (Shapiro and Hotchkiss, 1996). The importance of NO in the modulation of gastric ulcer healing had recently been elucidated (Sistani Karampour et al., 2019). Administration of NO donors can hasten the healing of a gastric ulcer (El-Abhar, 2010). In this experimental study, the levels of gastric PGE_2_ and NO were markedly elevated in rats treated with pomegranate peels, both in the natural extract formula and in bound form to chitosan nanoparticles. These results were in compliance with the previous findings (Sistani Karampour et al., 2019).

In the present study, we confirmed this latter finding where the PPE treated group showed a marked upregulation of eNOS mRNA expression and prominent positive eNOS immunostained cells in the vascular endothelium, surface epithelium, and glandular epithelial cells of the stomach mucosa, thereby replenishing the reduced eNOS found in the IND-treated group. This wide expression in several areas ensures enough supply of NO. This result was ascertained by the positive relationship between NO and PGE_2_ in PLN-treated rats and negative correlations between NO, each ulcer index, and MDA in PPE-treated groups.

The ability of pomegranate to promote local gastric defense mechanisms as well as re-epithelialization and regeneration of glandular architecture of stomach mucosa observed in the histological sections is attributed to its active ingredients. The polyphenolic constituents of pomegranate stimulate the formation of tissue growth factors, prostaglandins, eNOS-mediated NO generation, boosting endogenous antioxidant mucosal status, chelating oxidative agents, and suppressing anti-angiogenic factors (Al-Rehaily et al., 2002). Furthermore, tannins, another beneficial component of PPE, promoted healing of the ulcer by stimulating the precipitation of microproteins at the ulcerative site, thus forming a protective layer against irritants over gastric mucosa and inhibiting gastric secretions at the injury site (Chang et al., 2005). It was postulated that the antioxidant capacity of prepared pomegranate rind extract in methanol is greater than the seeds. The polyphenolic contents of pomegranate are higher when compared with different fruits such as an apple, grapefruit, pineapple, grapes, and an orange (Chidambara Murthy et al., 2002; Raja et al., 2007).

Moreover, the alkaloids constituents of PPE increase the pH of gastric contents by reducing HCL secretion, while flavonoids are capable of chelating free radicals due to the lack of electrons in their chemical structure, thus ameliorating oxidative injury to gastric mucosa (Ramamurthy and Marueen, 2018). Another study conducted by Ansar et al. (2019) reported that the antioxidant activity of PPE might be similar to curcumin due to the presence of the hydroxyl (- OH) group, that prohibits oxidative breakage of the -SH group, thus, preserving the thiol content of tissues protecting against oxidation of cellular proteins.

In the field of nanotechnology, a fantastic breakthrough in the science of improving the healing properties of the ulcerhas been made (Lim et al., 2016). Nanoparticles are a drug delivery tool that can be used either as a drug carrier or as the treatment itself (Ghosh et al., 2012). In this study, the nanosized pomegranate particles ranged in size between 299 and 900 nm with a PDI from 0.2 to 0.5. The nanoparticle size decreased when increasing the CS/TPP ratio, which might be due to the increase in the amount of chitosan, thus increasing the electrostatic interactions between chitosan and pomegranate. The pomegranate-loaded nanoparticles had a spherical shape and encapsulation efficiency of about 85 ± 0.6%, with positive surface charges due to the presence of excess chitosan functional amine groups. These positive surface charges of PLN were crucial in determining the electrostatic interactions and adhesion characteristics with the gastric mucosa. It was reported by Salama et al. (2019) that the transport of nanoparticles that have positive charges through epithelial cellular membranes was greater than the molecules that have no or negative charges, hence, enhancing their bioavailability.

Additionally, the stability study of PLN confirmed that the nanoparticles had sufficient stability over a long incubation period. These results established the role of TPP as a stabilizer as well as its ability to form strong electrostatic interactions and to form stable structures that are less able to aggregate and coalesce (Raja et al., 2015). This explains the effectiveness of PLN in curing the gastric ulcer, proven by the full restoration of biochemical, genetic, histopathological, and immunohistochemical alterations when compared with PPE-treated rats.

In conclusion, PLN and PPE can potently correct the harmful effects of IND on the gastric mucosa. This effect is more pronounced with PLN; the antiulcer activity of PPE can be ascribed to oxidant lowering and anti-inflammatory actions that in turn promote gastric protective factors (NO, PGE_2_), enhance stomach eNOS mRNA and protein expression, and markedly ameliorate all histopathological alterations. This study, therefore, offers new therapeutic agents for IND-induced gastric ulcers. However, future research is recommended to further explore the exact mechanism of pomegranate nanoparticles and to clarify their safety levels.

## Data Availability Statement

The raw data supporting the conclusions of this article will be made available by the authors, without undue reservation.

## Ethics Statement

The animal study was reviewed and approved by the Ethics Committee at the College of Medicine, Assiut University, Assiut, Egypt.

## Author Contributions

NA and MD conceived and designed the study, supervised conducting the experiment, analyzing the data and results, writing the first draft of the manuscript, and did critical reading of the manuscript then revising of manuscript in its submitted form. NA submitted the manuscript. MD performed the biochemicals and molecular expression tests, applying for fund and ethical committee acceptance. AA, DE, and AF shared the design of the study, performed the histopathological studies, analyzed the results, and shared in writing the manuscript. SH performed the nanoparticle. MA performed the pomegranate extract. ZS shared in writing the manuscript, and critical reading the manuscript from the clinical point of view. DA and AA involved in researched literature, conceived the study, help in writing the first draft of the manuscript. AA and MK involved in researched literature, conceived the study, help in writing the first draft of the manuscript. HG conceived the study, supervised conducting the experiment, shared in analyzing the data, shared in writing, and critical reading of the manuscript. All authors reviewed and edited the manuscript and approved the final version of this manuscript.

## Conflict of Interest

The authors declare that the research was conducted in the absence of any commercial or financial relationships that could be construed as a potential conflict of interest.
